# Cardiogenic shock revealing myocarditis after mRNA vaccination against covid-19: Case report and brief review for the first case in Morocco

**DOI:** 10.1016/j.amsu.2021.103210

**Published:** 2021-12-30

**Authors:** Hamza Mimouni, Choukri Bahouh, Saida amaqdouf, Ilyass laaribi, Mohammed Baddi, Samya Berichi, Houssam Bkiyar, Nabila ismaili, Noha El ouafi, Brahim Housni

**Affiliations:** a Faculty of Medicine and Pharmacy, Mohammed I^st^ University, Oujda, Morocco; b Department of Anesthesiology and Intensive Care Unit, Mohammed VI University Hospital Mohammed I University, Oujda, Morocco; c Department of Cardiology, Mohammed VI University Hospital Mohammed I University, Oujda, Morocco; d Mohammed First University, Faculty of Medecine and Pharmacy, LAMCESM, Oujda, Morocco; e Mohammed First University, Faculty of Medecine and Pharmacy, LERCSP, Oujda, Morocco

**Keywords:** Cardiogenic shock, Myocarditis- Covid-19, mARN vaccination

## Abstract

**Introduction:**

and importance: After its unexpected effectiveness in the clinical trials, the anti-COVID-19 vaccine type mRNA was launched on December 11, 2020, but a few months later, several reports of post-mRNA vaccination myocarditis were published, but without any proven causal link.

**Case presentation:**

We report the case of a 14-year-old teenager admitted to the emergency department for a cardiogenic shock, the patient mentioned that he had an anti-COVID 19 vaccination 10 days before his admission. First, the vasoactive drugs had stabilized the patient; the troponins came back highly favorable but later confirmed myocarditis by magnetic resonance imaging. In this sense an etiological analysis was made and it came back without any particularities, leaving us relating the myocarditis to the vaccination.

**Clinical discussion:**

Post-vaccination myocarditis is a rare event, with very few reports in the literature. After the introduction of COVID vaccination, several reports were published, mostly after the mRNA vaccine. Until now, no causal link has been proven, so we need to have more reports in this sense to have a better knowledge of this phenomenon.

**Conclusion:**

Until we obtain a more precise explanation of the mechanism of myocarditis after vaccination with the anti-COVID-19 vaccine, all symptoms suggesting myocarditis should be systematically monitored during the first 7 days after vaccination.

## Highlights

• Myocarditis after COVID 19 vaccination is currently a controversial issue.
• Cardiac MRI is the gold standard for diagnosis.
• Cardiogenic shock is a serious complication of acute myocarditis.

## 1 Introduction

Since April 2021, several cases of diseases including myocarditis have been reported after having COVID-19 vaccination type mARN [1], Most of myocarditis conditions were reported in young people, but to date, there is no proven causal link. We report the case of a young patient admitted with myocarditis complicated by cardiogenic shock in whom the etiology was related to the anti-COVID-19 vaccination type mARN.

## 2 Case presentation

We report the case of a 14-year-old child, without any pathological history, admitted to the emergency room for extreme asthenia associated with a headache and fever reaching 40 C°, resistant to the usual analgesics. The initial examination found a conscious patient, without deficit, with a frank stiffness of the neck, hemodynamically unstable with a SBP (systolic blood pressure) 80mmhg, and a DBP (Diastolic blood pressure) 30mmhg, tachycardia at 132bpm, with coldness of the extremities, on the respiratory level the patient was polypneic with a FR at 29cpm and SpO2 at room air 90%. The clinical examination showed signs of right heart failure, including turgidity of the jugular veins and hepatomegaly, without skin signs in favor of purpura. The ECG showed sinus tachycardia without repolarization disorders, and the chest X-ray in front of the patient's bed showed an alveolar syndrome.

**Fig. 1:**
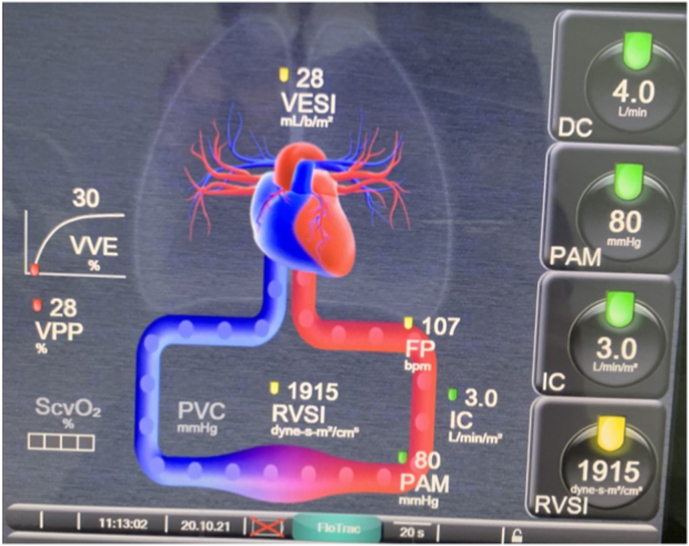
Monitorage hémodynamique invasif par système hemosphere edwards.

The patient was admitted to the intensive care unit equipped with a central femoral venous line and a right radial arterial line with invasive hemodynamic monitoring by pulse wave analysis through the hemosphere advanced system with results in favor of a cardiac component of the shock with a Cardiac Output of 3.4 l/min, a Cardiac Index of 1.9 l/min/m2 and normal peripheral vascular resistances *Trans*-thoracic echocardiography showed global hypokinesia (Supplementary material) with severe left ventricular systolic dysfunction with an ejection fraction of 25%, and the patient was then hemodynamically stabilized with the introduction of norepinephrine at a dose of 0.3ug/kg/min and dobutamine at a dose of 5ug/kg/min with a MAP of 80mmhg and Cardiac Output of 4l/min (Fig. 1).

A biological assessment was performed, showing a major elevation of ultrasensitive troponins to 1082.8 pg/ml (Normal value 0–26 pg/ml), natriuretic peptides to 11335, 8 pg/ml (Normal Value 0–100pg/ml), C-reactive protein at 296,11mg/l (Normal Value 6–12mg/l), blood creatinine at 22,35mg/l (Normal Value 0–12 mg/l), Urea at 2,49g/l (Normal value 0,35-0,55g/l). The infectious check-up including a lumbar puncture with study of the cerebrospinal fluid came back without particularities.

**Fig. 2:**
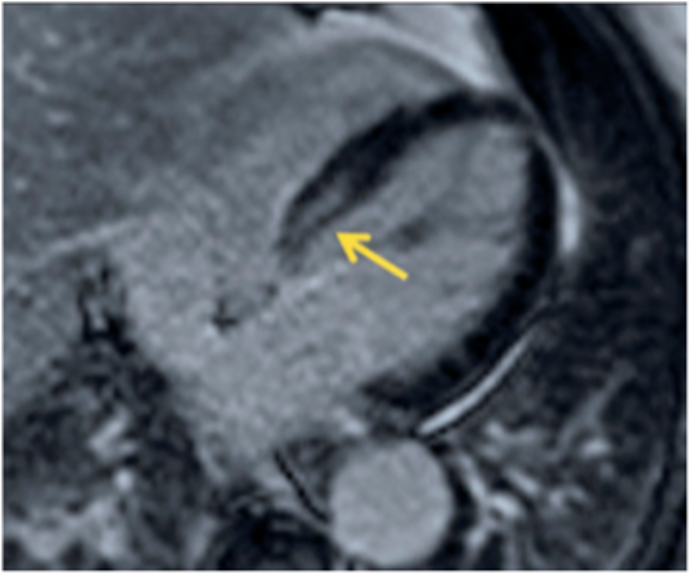
Cardiac MRI showing evidence of myocarditis.

Given the age of the patient, the clinical presentation on admission, the results of the echocardiography and the very high level of cardiac enzymes, the diagnosis of myocarditis was suspected. A cardiac MRI was consequently ordered, and it came back in favor of myocarditis (Fig. 2). On day 2 of the hospitalization, the patient was weaned from Norepinephrine with a low dose of dobutamine maintained. As for the etiology, the patient did not mention any signs of an influenza syndrome before his admission, the viral serologies requested were negative, the complete clinical examination did not reveal any systemic disease, and the eosinophil count was normal. However, the patient was vaccinated against COVID-19 with the mARN vaccine 10 days before his admission. In the light of this vaccination experience, the literature reports, as well as the clinical and paraclinical invistigations, the diagnosis of post-vaccination myocarditis is retained. Treatment with corticoids was started.On day 6 of his admission, the patient was completely weaned from vasoactive drugs, with an echocardiographic improvement of the left ventricular ejection fraction FE = 55% (Supplementary material. The patient was discharged home at Day 7 with good clinical and biological improvements. On the therapeutic level, corticosteroid treatment was maintained, with low doses of conversion enzymes.

## 3 Discussion

Given the complicated pathophysiology of the disease and its polymorphism which are mainly represented by the lung and the heart [[2], [3], [4]] COVID-19 infection has become a real challenge for all health systems to manage after its declaration as a global pandemic.

Considering the gravity of this disease, and the number of deaths that it has caused, as well as the ineffectiveness of the majority of the therapies used, the world was in need of an effective vaccine, without any major complications, and within a short period of time to control this famous pandemic.

On December 11, 2020, the Food and Drug Administration (FDA) issued an Emergency Use Authorization (EUA) for the Pfizer-BioNTech COVID-19 mRNA vaccine for the prevention of COVID-19 in people who are 16 years old and above [5]. On May 10, 2021, the FDA revised the EUA for this vaccine to include children 12 years and older. As the only vaccine approved for children 12–17 years of age [6]. The efficacy of mRNA vaccine in preventing COVID-19 infection in participants aged 16–55 years is 94–95%, and is 100% in children aged 12–15 years [7].

It should be noted that myocarditis after vaccination is a rare event, and has been described in some vaccines such as the smallpox vaccine [8] . Concerning the vaccination against COVID-19, several reports were published in this sense, mostly after a mRNA vaccine [9].

Regarding the physiopathology, there is no causal link yet demonstrated, but it is already noted in Pfizer-BioNTech clinical trials that systemic reactogenicity and immunogenicity are increased after the vaccine, especially for the young people [5], this reactogenicity is mainly made of extreme asthenia, myalgias, fever, arthralgia or lymphandinitis [6] , and maybe the myocarditis would be explained by this reactogenicity situation.

The symptomatology of myocarditis is varied, ranging from simple shortness of breath, chest pain, or dyspnea to the fulminant myocarditis, in which the patient comes with a shock that usually requires circulatory support with positive inotropes. Regarding myocarditis after mRNA vaccination, according to the Center for Disease Control and Prevention it is recommended to monitor the signs within 7 days after vaccination [9].

The first steps in the diagnosis of myocarditis are the anamnesis and the electrocardiographic signs, which are generally non-specific, and which may show arrhythmia or repolarization disorders [11]. The biology may show an elevation of markers of inflammation including C-reactive protein, and an increase in the troponin levels, but it should be noted that a normal troponin level does not eliminate the diagnosis. Echocardiography is recommended in all patients suspected of having myocarditis, it helps us evaluate the regional and global function of the left ventricle, to estimate the ejection fraction, and it should be pointed out that a normal ejection fraction should not exclude the diagnosis of myocarditis [12] . Cardiac magnetic resonance imaging (CMR) remains the examination of choice for diagnosis, it allows a precise characterization of the myocardial tissue. The criteria lake louise is used to identify the type of myocardial damage [13].

The therapeutic management of myocarditis after COVID-19 mRNA vaccine is essentially based on two elements: the first is the management of the hemodynamic instability by vasoactive inotropes or circulatory support such as the extracorporeal membrane oxygenation (ECMO), or the intra-aortic counterpulsation (IABP) in the fulminant forms [10], the second is the management of the inflammation, corticosteroids and intravenous immunoglobulins (IVIG) are used to limit the activation of the nonspecific immune system [14]. For non-steroidal anti-inflammatory drugs, it is recommended to not use them due to their detrimental action on the kidney, which may aggravate the heart failure by the significant hydro-sodic retention [15].

In our case, the hemodynamic instability was managed with vasoactive drugs only, and the use of corticosteroids alone was advocated to combat immune activation, and the evolution was favorable.

The SCARE guidelines were used in the writing of this paper [16].

## 4 Conclusion

Given the seriousness of this disease, which can be fatal in its fulminant form, further research on the causal links between myocarditis and mRNA type COVID-19 vaccination is needed, especially in view of the large number of cases reported.

## Ethical approval

The ethical committee approval was not required give the article type (case report).However, the written consent to publish the clinical data of the patients was given and is available to check by the handling editor if needed.

## Sources of funding

This research did not receive any specific grant from funding agencies in the public, commercial, or not-for-profit sectors.

## Author contribution

HAMZA MIMOUNI: study concept or design, data collection, data analysis or interpretation, writing the paper, Choukri Bahouh: Data collection, data analysis, Saida amaqdouf: Data collection, data analysis, Ilyass laaribi: Data collection, data analysis, Mohammd Baddi: Data collection, data analysis, Samia berrichi: Data collection, Houssam bkiyar: supervision and data validation, Brahim Housni: supervision and data validation.

## Registration of research studies

This is not an original research project involving human participants in an interventional or an observational study but a case report. This registration is was not required.

## Guarantor

HAMZA MIMOUNI.

## Consent

Written informed Consent was obtained from the child's parents for publication of this case report and accompanying images. A copy of the written consent is available for review by the Editor-in-Chief of this journal on request.

## Provenance and peer review

Not commissioned, externally peer reviewed.

## Declaration of competing interest

The authors state that they have no conflicts of interest for this report Ethical Approval The ethical committee approval was not required give the article type case report. However, the written consent to publish the clinical data of the patients were given and is available to check by the handling editor if needed.
